# Inhibition of Cellular and Animal Inflammatory Disease Models by NF-κB Inhibitor DHMEQ

**DOI:** 10.3390/cells10092271

**Published:** 2021-09-01

**Authors:** Jun Ma, Yuyang Zhang, Takeshi Sugai, Tetsuo Kubota, Hiroshi Keino, Magdy El-Salhy, Michitaka Ozaki, Kazuo Umezawa

**Affiliations:** 1 Shenzhen Wanhe Pharmaceutical Co., Ltd., Shenzhen 518107, China; majun@wanhe-phar.com; 2 School of Life Science and Biopharmaceutics, Shenyang Pharmaceutical University, Shenyang 110016, China; 13614053862@163.com; 3 Faculty of Pharmacy, Keio University, Tokyo 105-8512, Japan; sugai-tk@pha.keio.ac.jp; 4 Department of Medical Technology, Tsukuba International University, Tsuchiura 300-0051, Japan; kbtmtec@tmd.ac.jp; 5 Department of Ophthalmology, Kyorin University School of Medicine, Tokyo 181-8611, Japan; hkeino@ks.kyorin-u.ac.jp; 6 Department of Medicine, Stord Helse-Fonna Hospital, Tysevegen 64, 54 16 Stord, Norway; magdy.el-salhy@helse-fonna.no; 7 Department of Biological Response and Regulation, Faculty of Health Sciences, Hokkaido University, Sapporo 060-0812, Japan; ozaki-m@med.hokudai.ac.jp; 8 Department of Molecular Target Medicine, Aichi Medical University, Nagakute 480-1195, Japan

**Keywords:** NF-κB, DHMEQ, tacrolimus, inflammation, transplantation, ointment, intraperitoneal administration

## Abstract

General inflammatory diseases include skin inflammation, rheumatoid arthritis, inflammatory bowel diseases, sepsis, arteriosclerosis, and asthma. Although these diseases have been extensively studied, most of them are still difficult to treat. Meanwhile, NF-κB is a transcription factor promoting the expression of many inflammatory mediators. NF-κB is likely to be involved in the mechanism of most inflammatory diseases. We discovered a specific NF-κB inhibitor, dehydroxymethylepoxyquinomicin (DHMEQ), about 20 years ago by molecular design from a natural product. It directly binds to and inactivates NF-κB components. It has been widely used to suppress cellular and animal inflammatory disease models and was shown to be potent in vivo anti-inflammatory activity without any toxicity. We have prepared ointment of DHMEQ for the treatment of severe skin inflammation. It inhibited inflammatory cytokine expressions and lowered the clinical score in mouse models of atopic dermatitis. Intraperitoneal (IP) administration of DHMEQ ameliorated various disease models of inflammation, such as rheumatoid arthritis, sepsis, and also graft rejection. It has been suggested that inflammatory cells in the peritoneal cavity would be important for most peripheral inflammation. In the present review, we describe the synthesis, mechanism of action, and cellular and in vivo anti-inflammatory activities and discuss the clinical use of DHMEQ for inflammatory diseases.

## 1. Introduction

Nuclear factor-κB (NF-κB) was discovered about 35 years ago as a transcription factor interacting with the immunoglobulin enhancer sequences [1]. Later it was shown to promote the expression of many inflammatory mediators and apoptosis inhibitory proteins [2]. Therefore, it has been considered to be an attractive molecular target of inflammation therapy and cancer therapy. However, it has been difficult to find effective NF-κB inhibitors for the development of either an anti-inflammatory or anticancer agent. The reason is that NF-κB inhibitors should theoretically cause serious side effects, such as bone marrow toxicity. Indeed, NF-κB promotes essential factors, such as GM-CSF [3] and M-CSF [4] for blood cell differentiation.

**Figure 1:**
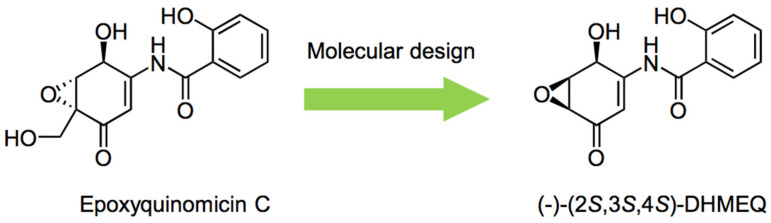
Molecular design of DHMEQ based on an antibiotic epoxyquinomicin C.

Meanwhile, dehydroxymethylepoxyquinomicin (DHMEQ) is a low molecular weight inhibitor of NF-κB, and its unique mechanisms of action have been elucidated. About 20 years ago, a novel epoxyquinone compound called epoxyquinomicin C (Figure 1) was isolated from the microorganism *Amycolatopsis* as a weak antibiotic [5]. Although it was structurally related to panepoxydone [6] and cycloepoxydone [7] that were reported to inhibit NF-κB, epoxyquinomicin C did not inhibit NF-κB. However, after the removal of the protruding hydroxymethyl moiety, the designed compound, dehydroxymethylepoxyquinomicin (DHMEQ, Figure 1), was found to inhibit NF-κB activity [8]. It inhibited TNF-α-induced activation of NF-κB activity in human T-cell leukemia Jurkat cells in the κB-luciferase system. DHMEQ also ameliorated collagen-induced rheumatoid arthritis in mice by IP administration [8]. Moreover, the original compound, epoxyquinomicin C, did not show any toxicity in animals, and fortunately, the designed compound, DHMEQ, exhibited the same results.

In the present review, we explain the discovery, synthesis, and mechanism of action. Then, we summarize the anti-inflammatory activities of DHMEQ in cellular and animal experiments. Finally, the future clinical application of DHMEQ is discussed.

## 2. Preparation of Racemic and Enantiomerically Pure DHMEQ

**Figure 2:**
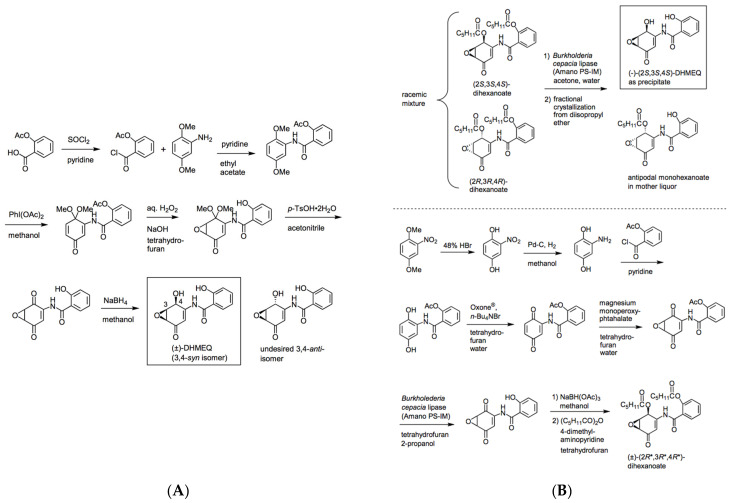
(**A**) Preparation of racemic DHMEQ. (**B**) Chemo-enzymatic synthesis of enantiomerically pure DHMEQ.

Racemic DHMEQ can be synthesized from 2,5-dimethoxyaniline in five steps [8,9]. The latest version of racemic DHMEQ synthesis is shown in Figure 2A. It is now possible to prepare DHMEQ with over 99.5% purity. In the final step, the desired active 3,4-*syn* isomer formed highly predominantly over the undesired 3,4-*anti* isomer. In most cases, substituted aniline is reacted with acetylsalicyloyl chloride to form an amide bond at the start [8,9]. But we also developed another route in which the amide formation is carried out at a later stage [10]. This route is useful to prepare various DHMEQ analogues. The structure-activity relationship study has revealed that the hydroxyl group at the 2-position of benzamide moiety is essential for inhibitory activity [10].

Thus, highly purified racemic DHMEQ can be effectively prepared using inexpensive chemicals.

The absolute stereochemistry of DHMEQ was determined by comparing the circular dichroism of (+)-DHMEQ with that of 1-epiepoxyquinomicin C. As a result, (+)-DHMEQ was shown to be (2R, 3R, 4R)-DHMEQ, while (−)-DHMEQ to be (2S, 3S, 4S)-DHMEQ [8]. Each enantiomeric form was firstly separated by HPLC with chiral stationary phase, and (−)-DHMEQ was shown to be about 10 times more effective than (+)-DHMEQ in inhibiting NF-κB [9]. At present, (−)-DHMEQ can be more effectively prepared by chemo-enzymatic synthesis with lipase-catalyzed kinetic resolution (Figure 2B). Commercially available *Burkholderia cepacia* lipase (Amano PS-IM, an immobilized form) reacts with racemic dihexanoylated form of DHMEQ to give (−)-DHMEQ in enantiomerically pure state and monohexanoylated form of undesired (2*R*,3*R*,4*R*)-antipode that can be easily removed by the difference in solubility. An alternative route to serve this substrate efficiently for lipase-catalyzed kinetic resolution was developed. In this synthesis, crude DHMEQ was directly acylated and racemic, but a distereomerically pure form of dihexanoate was obtained [11].

We also found an effective way of (+)-DHMEQ preparation with *Burkholderia cepacia* lipase-catalyzed hydrolysis of structurally modified precursor [12]. Moreover, we found that (+)-DHMEQ activated Nrf2, which is a transcription factor that induces the expression of multiple antioxidant enzymes in human neuroblastoma cells. It activated Nrf2 in a promoter-reporter assay and increased the expression of antioxidant proteins to inhibit ROS-mediated cell death in the neuronal cell line [12]. Inhibition of NF-κB can induce NRF2 [13]. However, (+)- and (−)-DHMEQ are equally active to induce NRF2 [12], although (+)-isomer is about 10 times weaker than (−)-isomer in inhibition of NF-κB. Therefore, it is unlikely that (+)-DHMEQ induces NRF2 via NF-κB inhibition. On the other hand, racemic DHMEQ was reported to produce ROS to explain the inhibition of NF-κB [14], and the ROS generator can induce NRF2 [12]. Therefore, it is possible that (+)-DHMEQ would induce NRF2 by the production of ROS.

Thus, enantiomerically pure DHMEQ can be prepared by chemo-enzymatic synthesis. (−)-DHMEQ has been mainly used for mechanistic study. At present, racemic DHMEQ is mainly used for basic study and the development of drugs.

## 3. Mechanism of NF-κB Inhibition by DHMEQ

### 3.1. Direct Inactivation of NF-κB Components

**Figure 3:**
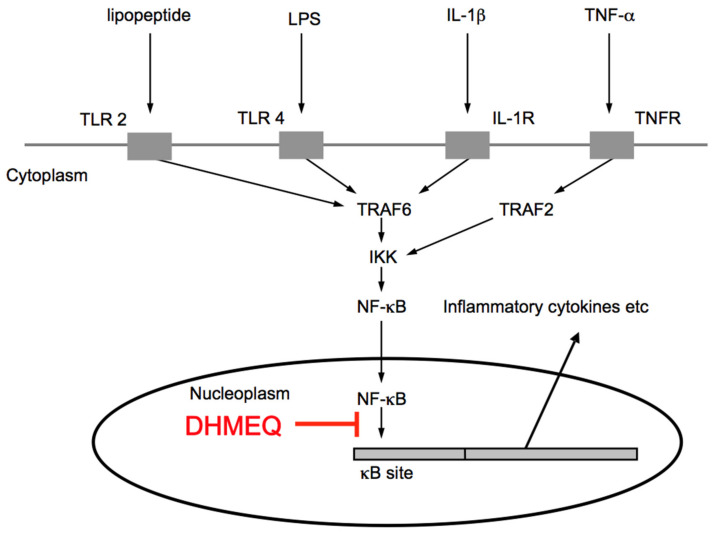
Signaling pathway for NF-κB activation and inhibition by DHMEQ.

For the inhibitory mechanism of DHMEQ, we first reported that it would inhibit the nuclear translocation of NF-κB [15]. However, we later found that DHMEQ covalently binds to the Rel-family proteins to inhibit their DNA-binding activity [16]. Inhibition of NF-κB nuclear translocation is likely to result after the inhibition of DNA binding [17]. Thus, DHMEQ inhibits the last step of the process of NF-κB activation (Figure 3).

Typical NF-κB is a heterodimer consisting of two Rel family proteins. Rel family proteins include p65, RelB, c-Rel, p50, and p52. (−)-DHMEQ was found to bind to p65 covalently with a 1:1 stoichiometry as revealed by surface plasmon resonance and MALDI-TOF mass spectrum (MS) analyses. MS analysis of the chymotrypsin-digested peptide suggested the binding of (−)-DHMEQ to a specific cysteine residue. In the case of p65, DHMEQ only binds to the Cys38 residue, which is located close to the DNA. Observation of the p65 and (−)-DHMEQ adduct in MALDI-TOF MS would suggest that (−)-DHMEQ-cysteine binding is a covalent one. The formation of DHMEQ-cysteine covalent binding in the protein was supported by chemical synthesis of the conjugate molecule in a phosphate buffer [16,18]. Since (−)-DHMEQ covalently binds to the cysteine residue in an NF-κB molecule, the inhibitory effect is irreversible [19]. Although (−)-DHMEQ is considered to bind to cysteine SH, it should not bind in a nonspecific manner. It is likely that DHMEQ enters into a specific pocket in the NF-κB component protein via a key and lock mechanism to bind to the limited cysteine residue.

**Figure 4:**
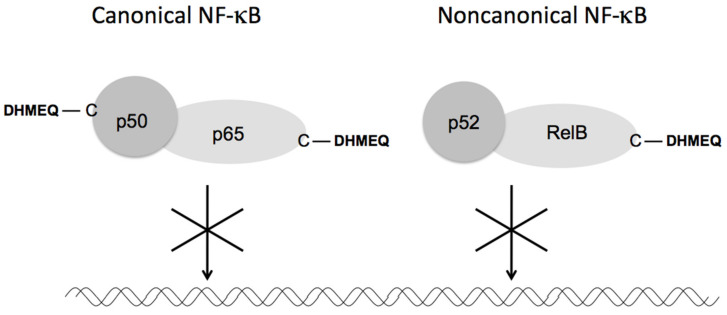
Inhibition of canonical and non-canonical NF-κB by DHMEQ. DHMEQ binds to specific cysteine residues in p65, p50, and RelB.

The signaling pathways for NF-κB activation include canonical and non-canonical ones [2]. The former consists mainly of p65 and p50 and is important for general inflammation and cancer progression, while the latter consists of RelB and p52 and is important for B-cell maturation and autoimmunity. All Rel-family proteins possess specific cysteine residues essential for their DNA binding. (−)-DHMEQ binds to these cysteine residues of p65 (Cys38), cRel (Cys27), RelB (Cys144), and p50 (Cys62), but not of p52 (Cys57). These specific bindings were all proved by the Cys-Ala mutation experiments [16]. In the case of RelB, (−)-DHMEQ inhibited the interaction with importin and decreased the stability in addition to the inhibition of DNA binding [20]. Thus, (−)-DHMEQ specifically binds to a cysteine residue in both the canonical (p65 and p50) and the non-canonical (RelB) components to inhibit both types of NF-κB (Figure 4).

Thus, the mechanism of inhibition has been elucidated. Specific binding of (−)-DHMEQ to each NF-κB component can explain specific inhibition of NF-κB by DHMEQ.

### 3.2. Alternative Mechanism; ROS-Mediated Inhibition of NF-κB

NF-κB is also known as a redox-sensitive transcription factor activated by reactive oxygen species (ROS). However, recent studies also suggested that ROS have the potential to repress NF-κB activity [21]. In addition to the mechanism in which DHMEQ directly binds to the NF-κB components, ROS-mediated inhibition has been proposed. Lampiasi, Cervello et al. observed that DHMEQ stimulated ROS production and that pretreatment of the cells with the antioxidant N-acetyl-L-cysteine (NAC) significantly reduced DHMEQ-induced ROS generation [14]. Accordingly, NAC reversed the DHMEQ-induced growth inhibition, caspase activation, and cell death in human liver cancer cells. Moreover, DHMEQ induced the expression of genes involved in the endoplasmic reticulum stress response (*GRP78*, *CHOP*, *TRB3*), which was also reversed in the presence of NAC. They suggested that DHMEQ antitumor effects would be mediated through ROS generation. Nakajima, Kitamura, et al. reported the pleiotropic potential of DHMEQ for NF-κB suppression with further mechanistic study [22]. They found that sustained exposure of renal tubular cells to DHMEQ blocked TNF-α- and IL-1β-induced TGF-β-activated kinase 1 (TAK1) phosphorylation, a crucial event for NF-κB activation upstream of IκB kinase. Pretreatment with ROS generator inhibited cytokine-induced TAK1 phosphorylation and NF-κB activation, and scavengers of ROS attenuated the suppressive effects of DHMEQ on TAK1 and NF-κB. They also found that DHMEQ caused the unfolded protein response (UPR) through the generation of ROS. UPR is also called endoplasmic reticulum (ER) stress response. DHMEQ caused selective induction of ER stress activating factor C/EBPβ, and knockdown of C/EBPβ attenuated the inhibitory effect of DHMEQ. These results suggest that DHMEQ inhibits NF-κB signaling via the generation of ROS and ER stress.

The specificity of NF-κB inhibition by DHMEQ is more easily explained by the direct inactivation of NF-κB components. However, both the direct inactivation and ROS generating mechanisms are likely to contribute to the inhibition of NF-κB signaling by DHMEQ.

## 4. Anti-Inflammatory Activity of DHMEQ

### 4.1. Cellular Anti-Inflammatory Activity

Excess macrophage activation is considered to be involved in most cases of inflammation. Therefore, we looked into the effect of DHMEQ on lipopolysaccharide (LPS)-induced macrophage activation in mouse monocytic leukemia RAW264.7 cells [23]. (−)-DHMEQ inhibited LPS-induced nuclear translocation of p65, iNOS expression, nitric monoxide (NO) production, and secretion of IL-6, IL-12, and IL-1β. It also inhibited LPS-induced histamine production and histidine decarboxylase (HDC) induction in RAW264.7 cells [24]. However, there is no κB site in the HDC promoter. Knockdown of the transcription factor C/EBPβ reduced the HDC expression in LPS-treated cells. Nuclear p65 enhances the activity of C/EBPβ acting as a cofactor in vitro [25], and (−)-DHMEQ can lower the nuclear amount of p65. Therefore, DHMEQ inhibits HDC expression by indirect suppression of C/EBPβ activity.

We also employed primary culture of mouse bone marrow-derived macrophages (BMM). DHMEQ inhibited NF-κB activation, iNOS expression, and NO production induced by LPS [26]. DHMEQ also inhibited the LPS-induced secretion of IL-6 and TNF-α. It inhibited COX-2 expression and prostaglandin E_2_ production. BMM can incorporate oxidized LDL to give rise to foam cells, and the (−)-DHMEQ-treated bone marrow cells did not take up oxidized LDL.

Microglia are the macrophages in the central nervous system, and they work for the host’s defense against microbial invasion in the brain. However, microglia are often over-activated by cytokines or injured neuronal cells. Excess activation of microglia may accelerate the progression of neurodegenerative diseases. In fact, activated microglia have been associated with Alzheimer’s disease, multiple sclerosis, stroke, and dementia. Stimulated microglia secrete various bioactive molecules, such as NO, reactive oxygen species, and inflammatory cytokines. DHMEQ inhibited LPS-induced NF-κB and secretion of TNF-α and IL-6 in a mouse microglial cell line [27].

Mast cells play a role in allergic reactions, rheumatoid arthritis, and skin inflammation. Stimulation with antigen and IgE is known to activate NF-κB in mast cells. We have studied the role of NF-κB on cellular migration in mast cell-like rat RBL-2H3 and mouse primary culture mast cells [28]. (−)-DHMEQ inhibited antigen/IgE-induced NF-κB activation and expression of its target genes, such as IL-6 and TNF-α. (−)-DHMEQ was found to inhibit in vitro invasion toward the antigen without any toxicity in the Matrigel chamber assay. Treatment with antigen/IgE increased MMP expression, which was inhibited by DHMEQ. An MMP inhibitor inhibited the invasion toward the antigen. Therefore, (−)-DHMEQ is likely to suppress mast cell migration via inhibition of NF-κB-mediated MMP-2 expression.

Thus, DHMEQ suppressed macrophage, microglia, and mast cell activities. Therefore, DHMEQ is likely to suppress natural immunity, which would be important for in vivo anti-inflammatory activities. Other cellular anti-inflammatory activities are shown in each disease model section.

### 4.2. Inhibition of Skin and Nasal Inflammations

Atopic dermatitis is a chronic inflammatory skin disease with an adverse impact on patient well-being. It has become increasingly prevalent in industrialized countries, where it now occurs in 10% to 20% of children and 1% to 3% of adults [29]. Corticosteroids are generally prescribed to control the symptoms; however, repeated use often causes severe skin atrophy and susceptibility to infection. Tacrolimus, a calcineurin inhibitor, has been developed for the treatment of atopic dermatitis to avoid the side effects associated with topical use of corticosteroids. However, refractory atopic dermatitis still remains even in patients treated with both corticosteroids and tacrolimus.

We studied the anti-inflammatory effect of DHMEQ ointment in several in vivo models of atopic dermatitis [29]. First, we examined whether DHMEQ suppressed the contact hypersensitivity response. DHMEQ (1 mg/mL in acetone) or tacrolimus (1% ointment) was applied to the same ear of 2,4,6-trinitrochlorobenzene (TNCB)-treated Balb/c mouse skin. The DHMEQ-treated ears showed significantly less ear swelling than the tacrolimus-treated ears. To determine whether DHMEQ has any therapeutic potential, we applied DHMEQ to atopic dermatitis-like lesions of NC/Nga mice and evaluated the progression of skin reactions. NC/Nga mice are the spontaneous mouse models of atopic dermatitis. The NC/Nga mice with moderate to severe lesions were topically applied with 1% DHEMQ in plastibase (5% polyethylene and 95% mineral oil), 0.1% tacrolimus ointment, or 0.12% betamethasone ointment daily for two weeks. Topical DHMEQ application significantly improved the severity of skin lesions compared with the ointment base as well as compared with topical treatment with the tacrolimus or betamethasone ointment. Improvement in clinical skin condition by DHMEQ was also confirmed by histologic observation, which showed amelioration of hyperkeratosis, acanthosis, and dermal edema compared with the ointment base treatment. At the affected skin sites, the number of eosinophils and mast cells was significantly lower in the DHMEQ-treated mice than that in the control mice. In this way, atopic dermatitis-lesions were ameliorated by topical application of DHMEQ ointment [29].

Atopic dermatitis lesions were also induced by the repetitive and alternative application of 2,4-dinitrochlorobenzene (DNCB) and oxazolone (OX) on ears in BALB/c mice [30]. The mice were then externally treated with DHMEQ ointment. In this model, DHMEQ also inhibited ear swelling and relieved clinical symptoms of the atopic dermatitis-like lesions. DHMEQ treatment significantly decreased DNCB/OX-induced epidermal thickness, the infiltration of inflammatory cells, and the count of mast cells in the histopathology examination. It also suppressed the elevated level of IgE in serum and the mRNA levels of IFN-γ, IL-4, and IL-13 in the ear tissues [30]. Next, stratum corneum of the ear skin was additionally stripped off with surgical tape before each challenge with DNCB/OX. The procedure using adhesive tape in preparation significantly accelerated the formation of the lesion, and this modified model should be more helpful for evaluating drug efficacy than the previous one. DHMEQ ointment showed anti-inflammatory activity again in this modified model [31]. For the mechanism of anti-inflammatory activity, DHMEQ inhibited gene expressions of IL-4, IL-13, and IFN-γ in the ear tissues in this model.

**Figure 5:**
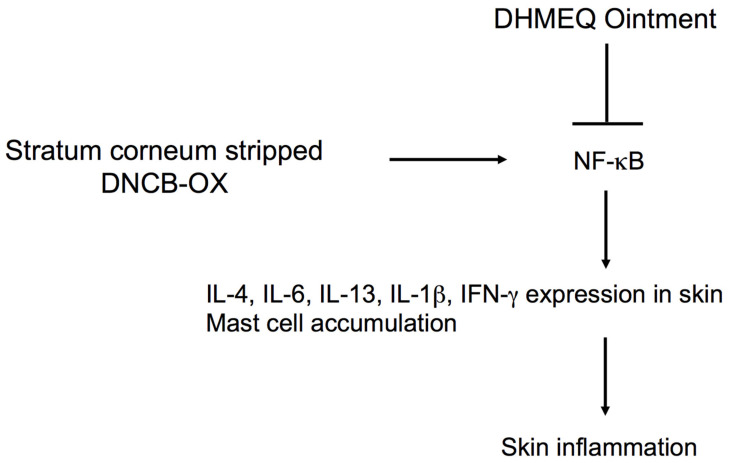
Inhibition of skin inflammation by DHMEQ ointment. DHMEQ directly inhibits NF-κB of skin inflammatory cells.

Using this model, the anti-atopic dermatitis activities of DHMEQ ointment were compared with that of tacrolimus ointment [32]. The mice were randomly divided into groups, which were normal, vehicle, DHMEQ (0.1%), and tacrolimus (0.1%). Most importantly, DHMEQ did not decrease body weight significantly. In contrast, tacrolimus led to a significant decrease in body weight after long-term application. Both of them significantly improved dermatitis symptoms of DNCB/OX-induced lesions, such as redness, itching, weeping, scaling, and thickening of the skin, while reducing epidermis thickness, dermis thickness, and the number of mast cells, as well. Both DHMEQ and tacrolimus similarly suppressed DNCB/OX-induced increase of serum IgE and attenuated expression of IL-4, IL-6, IL-13, IL-1β, and interferon (IFN)-γ in the disrupted ear tissues. In addition, the mice applied with tacrolimus became obviously irritable, jumping up and down, and inflammatory exudation on the skin surface was clearly observed. On the other hand, DHMEQ did not cause any adverse stimulus-response. Above all, the DHMEQ ointment is considered to have safer side effects and be similar in its effectiveness, and it would be more suitable for long-term use than the tacrolimus ointment [33]. Preparation of skin inflammation model in mice and amelioration by DHMEQ ointment is illustrated in Figure 5.

Keratinocytes are the major constituent of the epidermis and are actively involved in skin inflammatory responses by attracting leukocytes activating their functions. The increased keratinocyte activities are exerted in chronic skin inflammation, such as allergic contact dermatitis, psoriasis, and atopic dermatitis. For the study of cellular anti-inflammatory activity, Cardile, Malaponte, et al. employed the human keratinocyte NCTC 2544 cell line [33]. DHMEQ potently inhibited IFN-γ and histamine-induced ICAM-1, MCP-1, IL-8, and RANTES production at non-toxic concentrations. DHMEQ was stronger than hydrocortisone in inhibition of IL-8 and RANTES secretion.

Keloids are fibrous overgrowths due to an abnormal wound healing process after skin injury. They are characterized by the proliferation of dermal fibroblasts and an overproduction of the extracellular matrix. Keloids are a kind of benign tumor and do not regress with time. Surgical excision alone results in a high rate of recurrence, and treatment of keloids is one of the more challenging problems. Makino, Mitsutake, Yamashita, et al. employed primary normal and keloid fibroblasts for the study [34]. The NF-κB activity was constitutively elevated in keloid fibroblasts, indicating that NF-κB would be involved in keloid pathogenesis. DHMEQ markedly inhibited the NF-κB activity and reduced type I collagen accumulation in keloid fibroblasts at the non-toxic concentrations.

Chronic nasal polyposis characterizes the recruitment of inflammatory cells on the nasal mucosa, particularly eosinophils. In this process, fibroblasts, epithelial and endothelial cells are important producers of pro-inflammatory molecules, such as VCAM-1, ICAM-1, and RANTES. Migrated inflammatory cells produce other cytokines and chemokines, causing chronic inflammation. Topical glucocorticoid is the main choice for the initial treatment of nasal polyps. The glucocorticoid receptor acts as a negative transcription factor through the inhibition of other AP-1 and NF-κB expressions. Valera et al. described the higher expression of NF-κB in nasal polyps compared to normal nasal mucosa [35]. Moreover, they also showed that higher NF-κB expression would be correlated to a worse medical treatment outcome [36]. Therefore, NF-κB could be an important target for the treatment of inflammation and glucocorticoid resistance for nasal polyps. They employed DHMEQ to suppress inflammatory responses of nasal polyp fibroblasts prepared from the surgical tissue. DHMEQ inhibited TNF-α-induced nuclear translocation of NF-κB. Both the steroid fluticasone and DHMEQ inhibited VCAM-1, ICAM-1, and RANTES expression and secretion in nasal polyp fibroblasts [37]. Interestingly, DHMEQ showed synergistic effects with steroids in the anti-inflammatory activity. Thus, DHMEQ alone or with steroids may be a useful strategy to be explored, particularly for steroid-resistant nasal polyps.

DHMEQ ointment ameliorated various atopic dermatitis models in vivo without any toxicity. DHMEQ ointment is as active as and clearly safer than tacrolimus ointment. In addition, DHMEQ can inhibit inflammatory cytokine production not only in macrophage and macrophage-like cells but also in keratinocytes, keloid fibroblasts, and nasal polyp fibroblasts.

### 4.3. Amelioration of Rheumatoid Arthritis and Antiphospholipid Syndrome

#### Rheumatoid Arthritis

Rheumatoid arthritis is one of the most common connective tissue diseases featuring chronic polyarthritis. The etiology of rheumatoid arthritis remains uncertain. Many susceptibility genes have been reported, but no single gene was found to induce rheumatoid arthritis. Formerly, rheumatoid therapy depended primarily on corticosteroids and non-steroidal anti-inflammatory drugs to reduce pain, but significant prevention of the progression of bone destruction could not be achieved with such treatment. Newly developed biological agents, such as monoclonal antibodies against TNF-α, have demonstrated excellent efficacy in preventing joint damage, although there are some cases that are refractory to these biological agents. Further development of therapies that reduce not only inflammation but also bone destruction should be beneficial.

Wakamatsu, Kubota, et al. reported the therapeutic effects of DHMEQ on collagen-induced arthritis in mice [38]. Mice were treated with subcutaneous injections of DHMEQ daily from day 5 to 18 after the collagen treatment. Eighty percent of the mice showed paw swelling by day 5. Clinical assessment on day 18 revealed that DHMEQ treatment reduced the thickness of the paws and the number of swollen joints. Severe arthritis hampered the growth of young animals during the experimental period, but DHMEQ-treated mice showed less retardation of growth than controls. Furthermore, the DHMEQ group showed less soft tissue swelling and bone erosion on X-ray examination [39]. DHMEQ suppressed expression of CCL5, CCL2, IL-6, MMP-3, ICAM-1, VCAM-1 without showing cytotoxicity in cellular experiments using fibroblast-like synovial cells from the inflammatory site.

Takatsuna et al. reported that DHMEQ inhibited differentiation of mouse osteoclasts in cultured bone marrow-derived macrophage precursor cells in the presence of RANKL and M-CSF [40]. In those experiments, DHMEQ suppressed not only the activation of NF-κB but also the expression of NFATc1 that is known to be an essential transcription factor in osteoclastogenesis. This has been the first report that NFATc1 expression is regulated by NF-κB [40]. DHMEQ also suppressed the bone-resorbing activity of mature mouse osteoclasts. Then, the effect of DHMEQ on in vivo osteoclastogenesis was studied in the arthritis model [41]. Histochemical staining of the ankle joints at day 10 showed numerous TRAP-positive multinuclear giant cells (osteoclasts) along the inner surface of bone lacunae and bone erosion in vehicle-treated mice, while much smaller and less TRAP-positive cells were present in specimens from DHMEQ-treated mice [40]. The immunohistochemistry experiment revealed activation of DHMEQ treatment also inhibited NF-κB activity and expression of NFATc1 at the inflammatory site in mice. DHMEQ suppressed differentiation of osteoclasts without significant effects on expression of RANKL and M-CSF in cultured human monocytes. Similarly, Jimi et al. reported that a peptide inhibitor of the IκB-kinase suppressed mouse arthritis and osteoclastogenesis without modification of RANKL and osteoprotegerin [42]. Therefore, DHMEQ is considered to directly act on the osteoclast precursor cells rather than the RANKL producing cells.

When DHMEQ is injected into the subcutaneous cavity near the inflammatory site, as described above, it is considered to reach the joint and directly inhibit NF-κB of the inflammatory cells. In summary, inhibition of NF-κB pathways is effective for alleviating both inflammation and bone destruction in vivo and in situ, and it is proposed as a future therapeutic strategy for rheumatoid arthritis.

Cardile, Malaponte et al. set up a cellular model of arthropathy with chondrocytes, where oxidative-inflammatory stress induces the progressive destruction of the articular cartilaginous tissue [43]. Chondrocytes are the only cellular component of articular cartilage and are capable of phenotype modulation, making these cells pivotal in the progression of joint diseases, including rheumatoid arthritis. The primary culture of human chondrocytes was employed. DHMEQ inhibited IL-1β-induced iNOS and COX-2 gene expression while also suppressing the production of nitric monoxide. In addition, DHMEQ induces a significant decrease in ICAM-1 expression, in addition to MCP-1, RANTES, and IL-8 release. Subcutaneous administration of DHMEQ may be useful to ameliorate arthropathy caused by over-activation of chondrocytes.

Anti-phospholipid syndrome is an autoimmune disorder characterized by recurrent arterial or venous thrombosis and unexplained abortion or fetal death, with the presence of circulating anti-phospholipid antibodies. About half of the patients suffer from autoimmune diseases, mostly systemic lupus erythematosus. For the treatment of anti-phospholipid syndrome, antiplatelet agents and anticoagulant agents are commonly used, but recurrent events are difficult to prevent completely. β_2_-Glycoprotein I-dependent anti-phospholipid antibodies are considered to play an important pathogenic role in anti-phospholipid syndrome. The antibodies activate platelets via thrombin formation and suppress fibrinolysis. More recently, Kubota et al. proposed another possible mechanism that involves certain chemokines that would induce platelet activation [44]. This hypothesis is based on the observation that the antibodies stimulated monocytes to secrete inflammatory cytokines, such as IL-1β and TNF-α, which in turn stimulated vascular endothelial cells to express chemokines, such as CX3CL1 and CCL5. CX3CL1 promoted normal platelets to adhere to collagen, while CCL5 induced platelet aggregation. DHMEQ was found to inhibit the expression of IL-1β and TNF-α in the antibody-stimulated monocytes. DHMEQ also inhibited the expression of CX3CL1 and CCL5 by vascular endothelial cells stimulated with IL-1β and TNF-α. These results suggest that the NF-κB pathway may be a potential therapeutic target of anti-phospholipid syndrome [44].

Nishimura, Kubota et al. set up a unique in vivo model of anti-phospholipid syndrome by use of a dorsal skinfold chamber fitted to a BALB/c mouse, β_2_-glycoprotein I-dependent anti-phospholipid antibodies, and pulse-laser irradiation [45]. Using this model, anti-phospholipid antibodies-induced thrombus formation was ameliorated by IP administration of DHMEQ [45]. The mechanism of amelioration by IP administration may be different from that by subcutaneous administration, as discussed later.

### 4.4. Inhibition of Ophthalmic Inflammations

Uveitis is an inflammatory disorder of the uveal tract in the eye and can lead to visual impairment and blindness. Although topical and systemic corticosteroids have been used as standard therapy for uveitis, long-term use of corticosteroids can result in various adverse effects. Therefore, the development of alternative therapies to corticosteroids is expected. Ando, Keino et al. studied the effect of DHMEQ on endotoxin-induced uveitis in rats [46]. LPS was subcutaneously injected, and DHMEQ was administered in the peritoneal cavity. DHMEQ significantly reduced the number of infiltrating cells and the concentrations of TNF-α and IL-6 in the aqueous humor. In addition, isolated peritoneal exudate cells were exposed to LPS, with or without DHMEQ. DHMEQ suppressed the LPS-induced NF-κB activation and production of TNF-α, IL-6, and MCP-1 in these peritoneal exudate cells. Thus, DHMEQ was shown to inhibit a uveitis model in vivo and in vitro.

Iwata, Todo, Onoe, et al. employed experimental autoimmune uveoretinitis, a Th1/Th17 cell-mediated autoimmune disease induced in mice, as a model of human endogenous uveitis [47]. In this model, pro-inflammatory cytokines and various stimuli activate NF-κB in the retina. Experimental autoimmune uveoretinitis was induced by K2 peptide immunization. IP administration of DHMEQ delayed the onset of disease. Histologic severity was significantly milder in DHMEQ-treated mice than in controls. IP administration of DHMEQ also suppressed NF-κB translocation in the retina, which might have reduced the inflammation of ocular tissues.

The retinal pigment epithelium forms the outer retinal layer consisting of a monolayer of pigmented cells, and it maintains the integrity of the photoreceptors by phagocytosis and recycling of the retinal photoreceptor outer segments. The retinal pigment epithelium cells are the main source of inflammatory cytokines, including IL-6, MCP-1, and IL-8. ARPE-19 cells are typical, human retinal pigment endothelium cell lines, and they are often used to study the mechanism of inflammatory disorders, such as that of uveitis and age-related macular degeneration. Ando, Keino et al. reported DHMEQ inhibited expressions of IL-6, MCP-1, and IL-8 in this cell line [48]. Thus, DHMEQ showed an anti-inflammatory effect in a human retinal pigment endothelium cell line.

The mammalian target of rapamycin (mTOR) is an atypical serine/threonine-protein kinase that acts as phosphoinositide 3-kinase (PI3K). Rapamycin is an inhibitor of mTOR. Rapamycin is a clinically available immunosuppressant and is expected to be an anti-inflammatory drug for non-infectious ocular inflammatory disease and uveitis. Okamoto, Ozawa, Tsubota, et al. reported that rapamycin inhibited the post-transcriptional reduction in the visual pigment of rod photoreceptor cells and rod photoreceptor cell dysfunction during inflammation in mice [49]. It also inhibited activation of NF-κB and induction of inflammatory cytokines, such as IL-6 and MCP-1, and the activation of the downstream signaling protein STAT3, which reduces rhodopsin in the retina during inflammation. Increased leukocyte adhesion was also attenuated by rapamycin. Interestingly, although mTOR activation was observed after NF-κB activation, mTOR inhibition suppressed NF-κB activation at the early phase. Since the inhibition of NF-κB suppressed mTOR activation, there would be a positive feedback loop of mTOR and NF-κB during inflammation. DHMEQ was used in this research to elucidate the role of NF-κB. They also suggested that DHMEQ itself would protect eyes from inflammation.

AMP-activated protein kinase (AMPK) is known to regulate cellular energy homeostasis. An increase in the AMP/ATP ratio by starvation activates AMPK to phosphorylate key target proteins that induce energy production. AMPK is also modulated in response to inflammatory signals. Kamoshita, Ozawa, Tsubota, et al. investigated the role of activated AMPK in retinal neural damage and visual function impairment caused by inflammation [50]. For this purpose, they employed a mouse model of LPS-induced inflammation in the retina and examined the effects of an AMPK activator, 5-aminoimidazole-4-carboxamide ribonucleoside (AICAR). During inflammation, retinal AMPK activity was decreased, but AICAR prevented this decrease. Moreover, AICAR prevented visual dysfunction in this model. In parallel, AICAR suppressed activated NF-κB activity in the retina during inflammation. Treatment with DHMEQ preserved the rhodopsin level during inflammation, suppressing NF-κB. These findings indicated that AMPK activation and subsequent NF-κB inhibition had a protective effect on visual function. Again, DHMEQ itself is likely to protect eyes from inflammation.

Nagai, Ishida, et al. investigated the involvement of the renin-angiotensin system and the NF-κB pathway in diabetes-induced retinal inflammation [51]. Induction of diabetes in mice led to a significant increase in retinal expression and production of the renin-angiotensin system components, such as angiotensin II, AT1-R, and AT2-R. Retinal adherent leukocytes were significantly suppressed by angiotensin II signaling blockers. Administration of the angiotensin II signaling blocker inhibited diabetes-induced retinal expression of ICAM-1 and VEGF. DHMEQ also suppressed these cellular and molecular inflammatory parameters in the diabetic retina to the levels obtained with the angiotensin II signaling blocker treatment. DHMEQ was used to study the role of NF-κB in this study, but it was also shown that IP administration of DHMEQ would be useful to ameliorate diabetes-induced retinal inflammation.

Choroidal neovascularization is a pathogenic process of age-related macular degeneration, a vision-damaging disease. The retinal pigment epithelium and macrophages both influence the development of choroidal neovascularization. Hirasawa, Ozawa, et al. reported the role of angiopoietin-like protein 2, a cytokine involved in age-related systemic diseases on choroidal neovascularization [52]. Using a laser-induced choroidal neovascularization model, they found that angiopoietin-like protein 2-knockout mice exhibited lower disease development with reduced macrophage recruitment and inflammatory mediator production. Peritoneal macrophages derived from the knockout mice expressed lower levels of the inflammatory mediators. In the wild-type peritoneal macrophages, angiopoietin-like protein 2 induced the inflammatory mediators via integrins α4 and β2, followed by the downstream activation of NF-κB and ERK. The activation of NF-κB and ERK by angiopoietin-like protein 2 also promoted macrophage migration. Therefore, angiopoietin-like protein 2 from focal tissue might trigger macrophage recruitment, and that from recruited macrophages might promote expression of inflammatory mediators, including angiopoietin-like protein 2 in an autocrine and/or paracrine fashion to facilitate disease development. Although DHMEQ was used as a tool of mechanistic analysis in this study, its IP administration was shown to ameliorate choroidal neovascularization.

Inflammation of the corneal fibroblasts causes corneal scarring, neovascularization, ulceration, and vision reduction. Corneal fibroblasts can control local inflammation through the production of cytokines, chemokines, and adhesion molecules. Inokawa, Keino, et al. reported that DHMEQ inhibited IL-1β-induced IL-8 and MCP-1 production and ICAM-1 expression in primary culture of human corneal fibroblasts [53]. Thus, the application of DHMEQ may be useful to ameliorate corneal scarring.

Kubota, Tsubota, et al. reported the inhibition of ROS-induced angiogenesis in the cornea of mice by an antioxidant NAC and DHMEQ [54]. ROS production in the cornea increased immediately after alkali injury. Alkali injury also activated NF-κB and increased VEGF and MCP-1 production, leading to a larger area of angiogenesis. Angiogenesis in the cornea of SOD-1-deficient mice was higher than in wild-type mice. IP administration of DHMEQ or the antioxidant NAC significantly reduced corneal angiogenesis by inhibiting the NF-κB pathway in both wild-type and SOD-1-deficient mice. DHMEQ would be useful to reduce over-angiogenesis in eyes.

### 4.5. Inhibition of Digestive Tract Inflammation

Inflammatory bowel diseases, such as Crohn’s disease and ulcerative colitis, are chronic, relapsing inflammatory disorders of the gastrointestinal tract. The onset of inflammatory bowel diseases occurs mostly at a young age and causes lifelong illness. Treatments with 5-aminosalicylates and corticosteroids are beneficial for many inflammatory bowel disease patients but are not effective for most patients over the long term. The next generation of chemotherapy includes mercaptopurine, azathioprine, and methotrexate. However, both short- and long-term side effects limit their use. Biological agents, such as TNF-α antibodies, have been used, but the ratio of responsive patients is limited. Surgical treatment remains the only option for many inflammatory bowel disease patients. Therefore, new, safe, and effective therapy is expected.

El-Salhy et al. studied the anti-inflammatory effect of DHMEQ in rats with severe colitis induced by trinitrobenzenesulphonic acid (TNBS) [55]. DHMEQ was injected into the peritoneal cavity twice daily for 5 days. The body weight losses and mortality rates were significantly lower in the DHMEQ-treated group than in the control group. DHMEQ ameliorated all the inflammatory scores obtained from endoscopic scope, macroscopic appearance, and histopathological examination.

DHMEQ also ameliorated dextran sulfate sodium (DSS)-induced colitis in rats. In addition to the in vivo inflammatory parameters above, the submucosal densities of leucocytes, lymphocytes, macrophages/monocytes, and mast cells were lower in the DHMEQ-treated rats [56]. El-Salhy et al. also studied the effect of DHMEQ on the number of endocrine cells in the DSS-induced inflammatory bowel disease model in rats [57]. The levels of endocrine cells and immune cells were changed by treatment with DSS. IP administration of DHMEQ inhibited the change of both endocrine cells and immune cells. They concluded that there would be an interaction between endocrine and immune cells during the inflammation and that the changes in interaction would cause the clinical manifestation of colitis symptoms. The anti-inflammatory effect of DHMEQ was also reported by Salhy et al. on TNBS-induced colitis model in rats [58].

Funakoshi, Yamashita, Todo, et al. also showed anti-inflammatory activity of DHMEQ on either DSS- or TNBS-induced mouse colitis model [59]. DHMEQ was injected into the peritoneal cavity twice daily. DHMEQ significantly ameliorated DSS-induced colitis as assessed by disease activity scores, colonic edema, and histological scores. Furthermore, DHMEQ significantly ameliorated TNBS-induced colitis as assessed by body-weight changes and histological scores.

Intestinal ischemia and reperfusion lead to severe inflammatory responses in both local and remote organs, as in the lungs. Suzuki, Yamashita, Todo, et al. reported that IP administration of DHMEQ ameliorated intestinal ischemia in rats induced by occluding the superior mesenteric artery [60]. They found that the DHMEQ-treated animals exhibited higher values of intestinal tissue blood flow. DHMEQ suppressed TNF-α and IL-6 production, resulting in marked prolongation of the survival times. DHMEQ ameliorated severe intestinal mucosal damage and disruption of the lung alveolar architecture accompanied by hemorrhage and increased neutrophil infiltration.

Thus, IP administration of DHMEQ ameliorated inflammatory bowel diseases in various murine models. No side effects have been observed. Since there is no satisfactory therapy in this field, the use of DHMEQ may be a candidate for new treatment.

### 4.6. Inhibition of Renal Inflammation

Miyajima et al. studied whether DHMEQ would inhibit renal inflammatory responses in a rat unilateral ureteral obstruction model [61]. DHMEQ was administered into the peritoneal cavity of rats 1 day after unilateral ureteral obstruction and every day thereafter. Kidneys were harvested at day 7 after the unilateral ureteral obstruction. Unilateral ureteral obstruction significantly activated NF-κB, induced tubular apoptosis, tubular proliferation, and interstitial fibrosis in the obstructed kidney of the control group compared with their unobstructed counterparts. Daily administration of DHMEQ inhibited tissue NF-κB and these renal inflammatory markers in vivo.

NF-κB is known to be activated in mesangial proliferative glomerulonephritis, and anti-thy1.1 antibody-induced proliferative glomerulonephritis is often used for the model of this disease. Kosaka, Miyajima, Oya, et al. studied the amelioration of anti-thy1.1 antibody-induced glomerulonephritis in rats by DHMEQ [62]. DHMEQ treatment resulted in marked inhibition of tissue NF-κB, decreased proteinuria, preserved creatinine clearance, decreased glomerular cell proliferation, and mesangial matrix deposition, and an increase in glomerular and tubular apoptosis, without inducing any obvious adverse effects.

Minimal-change nephrotic syndrome is a kidney disease defined by proteinuria and hypoalbuminemia. NF-κB activation in glomerulus podocytes is considered to be associated with the development of this disease. Shimo, Adachi, Kaneko, et al. studied whether DHMEQ could ameliorate the nephrosis in mice induced by puromycin aminonucleoside, which is an animal model for minimal-change nephrotic syndrome [63]. Pretreatment with DHMEQ alleviated the proteinuria and reversed the serum abnormalities in this animal model. Increased serum IL-6 was completely suppressed by DHMEQ. Electron microscopic analyses of glomeruli indicated that DHMEQ recovered normal morphology of podocytes by inhibiting the NF-κB activity.

Cyclosporine A is a popular immunosuppressant used in organ transplantation. However, its chronic nephrotoxicity is an obstacle to long allograft survival. Morita, Shinoda, Oya, et al. investigated the effect of DHMEQ on cyclosporine A-induced nephrotoxicity in rats [64]. IP administration of DHMEQ inhibited NF-κB activation induced by cyclosporine treatment in the rat kidney tissue. Elevated serum urea nitrogen and creatinine levels due to repeated cyclosporine administration were also decreased by DHMEQ, and creatinine clearance was restored in the treatment group. The development of renal fibrosis due to chronic cyclosporine nephrotoxicity was significantly inhibited by DHMEQ treatment, and these observations reflected the results of renal functional assessment. DHMEQ also inhibited the elevated expression of MCP-1 and CCL5 and the accumulation of macrophages and granulocytes in the kidney tissue. These findings suggest that DHMEQ treatment in combination therapy with cyclosporine-based immunosuppression is beneficial to prevent the development of nephrotoxicity.

Thus, IP administration of DHMEQ ameliorated unilateral ureteral obstruction-induced renal inflammation and mesangial proliferative glomerulonephritis models in rats and minimal-change nephrotic syndrome models in mice. It also reduced the toxicity of cyclosporine A in the kidney. Clinical application of DHMEQ alone or with other therapy may be useful for the suppression of renal inflammation.

### 4.7. Inhibition of Graft Rejection in Organ Transplantation

Organ transplantation is one of the important regeneration therapies. However, it is often associated with immunological graft rejection, which limits the use of this therapy. The discovery of immune-suppressants such as tacrolimus and cyclosporine has made various organ transplantation possible, but there are still problems in their effectiveness and toxicity. IP administration of DHMEQ has been studied in various animal models of organ transplantation.

Ueki, Yamashita, Todo, et al. first reported inhibition of graft rejection by IP administration of DHMEQ in 2006 [65]. They employed a mouse cardiac allo-transplantation model. Single DHMEQ treatment moderately prolonged cardiac allograft survival. Further, the combination of DHMEQ plus tacrolimus markedly prolonged graft mean survival time. Such effects were associated with inhibition of mixed lymphocyte reaction against donor antigen, IFN-γ producing splenocytes, and graft cellular infiltration.

Goto, Yamashita, Todo, et al. evaluated the immunomodulatory effects of DHMEQ when combined with a donor-specific blood transfusion and assessed whether the treatment induces tolerance in a mouse heart transplantation model [66]. In fully mismatched H2-to-H2 heart transplants, donor-specific blood transfusion alone prolonged allograft median survival time to 15 days, whereas when combined with DHMEQ treatment, the graft median survival time was prolonged to 39.5 days. Thus, the combination of donor-specific blood transfusions and DHMEQ treatment may become useful therapy in organ transplantation.

Islet transplantation for insulin-dependent diabetes mellitus (type 1 diabetes mellitus) by the Edmonton protocol is widely used, but this therapeutic approach has a major limitation in that islets from two to four cadaveric pancreas are required to achieve insulin independence in one diabetic recipient. Large numbers of islet grafts are required mainly because of immune reactions, including inflammation that destroys islet grafts. Kuraya, Watanabe, Todo, et al. studied whether DHMEQ would ameliorate inflammatory responses after pancreatic islet transplantation [67]. One hundred seventy-five islets from C57BL/6 (B6) mice were transplanted into the liver of streptozotocin-induced diabetic B6 mice. The recipient mice were administered DHMEQ for 1, 2, or 3 days after the transplantation. With a vehicle treatment, only 11.1% of the islet recipients achieved normoglycemia. In contrast, DHMEQ treatment markedly improved to 83.3%, which was associated with the suppression of the serum level of high mobility group complex-1 (HMGB1). DHMEQ treatment also suppressed the expression of TNF-α, MCP-1, MIP-1β, IL-1β, and IL-6 in the transplanted liver. At the same time, Watanabe, Yamashita, Todo, et al. reported that the combination with tacrolimus markedly prolonged the graft survival [68]. A protocol with a 3-day treatment with DHMEQ, followed by a 2-week treatment with tacrolimus, allowed permanent acceptance of islet allografts.

The viability of islets is considered to be largely lost in the course of islet preparation from the donor. Therefore, the prevention of islet loss in the removal process of the pancreas is important for improving engraftment and for reducing the number of necessary islets. NF-κB is known to be relevant to the mechanism of β-cell apoptosis in isolated islets. Takahashi, Todo, et al. attempted to prevent islet apoptosis during isolation from the donor by DHMEQ in a mouse islet transplantation model [69]. NF-κB activity in islet was activated immediately after isolation, and IP administration of DHMEQ inhibited the NF-κB activation without deterioration of islet function. DHMEQ significantly prevented islet apoptosis by inhibiting caspase 3 and 7 activation and down-regulated Bax. Donor pretreatment with DHMEQ significantly improved engraftment in syngeneic islet transplantation in mice, thus preserving insulin contents in the graft liver. Thus, administration of DHMEQ in the peritoneal cavity of donors would be a useful strategy to reduce necessary islets for transplantation.

Allogeneic bone marrow transplantation is mainly used for the treatment of hematopoietic diseases, such as congenital immune deficiencies, aplastic anemia, and leukemia. But graft-versus-host disease (GVHD) is a crucial mortality factor in allogeneic bone marrow transplantation. Yamanouchi, Adachi, Kaneko, et al. showed that IP administration of DHMEQ suppressed GVHD, resulting in a decreased mortality rate in a mouse bone marrow transplantation model [70]. Bone marrow cells from C57BL/6 mice (B6 mice) were transplanted into lethally irradiated BALB/c mice. Two weeks later, spleen cells from B6 mice were transplanted into the irradiated BALB/c mice. From one week after the injection of spleen cells, when the mice started to show GVHD, DHMEQ was administered daily for 4 weeks. By 80 days after the transplantation, about 40% of the vehicle-injected mice died because of GVHD, whereas all DHMEQ-injected mice survived this observation period and developed milder GVHD than the vehicle-injected mice. When regulatory T cells were reduced, the effect of DHMEQ was reduced. Thus, DHMEQ is likely to be useful for the treatment of bone marrow transplantation.

Above all, DHMEQ IP administration can inhibit graft rejection in various organ transplantation models. Usually, DHMEQ is given to the recipients to prolong the survival of grafts. However, as in the case of islet transplantation above, administration of DHMEQ to the donor is also effective in prolonging the graft survival after the transplantation. Donor treatment therapy with DHMEQ may be useful in the future.

### 4.8. Amelioration of Endotoxin Shock, Asthma, and Other Inflammatory Diseases

Endotoxin shock is a fatal disease, but no effective therapy has been discovered. Asthma, atherosclerosis, and baby loss are also difficult to treat at present. Novel therapy is expected in these areas.

Endotoxin shock is caused by a bacterial infection-induced cytokine storm. Cell surface endotoxin LPS of Gram-negative bacteria stimulates inflammatory cells to release cytokines, such as TNF-α. Shimo, Adachi, Kaneko, et al. examined the effects of DHMEQ on LPS-induced TNF-α production in vivo and on the survival of mice [71]. When DHMEQ was intraperitoneally injected into mice 2 h before LPS injection, the survival of the LPS-injected mice was prolonged. When DHMEQ was injected twice (2 h before LPS injection and the day after LPS injection), all the mice were rescued. The injection of DHMEQ 1 h after LPS injection and the day after LPS injection also resulted in the rescue of all mice. DHMEQ lowered the serum levels of TNF-α in the LPS-treated mice. These results suggest that DHMEQ may be useful for the prevention and treatment of endotoxin shock.

Asthma is a chronic inflammatory disorder of the airways that is characterized by eosinophilic accumulation and inflammation, airway hyper-responsiveness, and airway remodeling. Shimo, Todo, Nishimura, et al. studied whether DHMEQ ameliorates airway inflammation and remodeling in murine models of asthma [72]. The BALB/c mice were sensitized and then challenged acutely or chronically with ovalbumin, and DHMEQ was administered intraperitoneally before each challenge. As a result, DHMEQ reduced the number of eosinophils and levels of IL-13 and IFN-γ in the bronchoalveolar lavage fluid. DHMEQ also inhibited parameters of airway remodeling, including mucus production, peribronchial fibrosis, and the expression of α-smooth muscle actin. Thus, DHMEQ may be useful for the treatment of asthma.

Atherosclerosis is a chronic inflammatory process, and anti-inflammatory agents inhibit the development of atherosclerosis. Chiba, Shimokado, et al. studied whether DHMEQ would ameliorate the in vivo atherosclerosis model [73]. DHMEQ was injected intraperitoneally into apoE-deficient mice 3 times a week for 16 weeks. The entire aorta was excised, and the atherosclerotic area was examined at 4 and 16 weeks. As a result, the atherosclerotic area was smaller in mice treated with DHMEQ, both at 4 and 16 weeks. Atherosclerosis lesions involve the adhesion of monocytes to the arterial intima, their exclusion from the vessel, differentiation into macrophages, and recruitment of smooth muscle cells. Therefore, the adhesion of monocytes to vascular endothelial cells is a critical step in atherosclerosis. Ohno, Kawai, et al. reported that DHMEQ inhibited the adhesion of monocytes to the human umbilical endothelial cells (HUVEC) under flow [74]. They used both freshly prepared HUVEC and human mononuclear cells. DHMEQ inhibited TNF-α-, IL-1β-, and LPS-induced NF-κB activation in HUVEC. It also inhibited the TNF-α-induced expression of ICAM-1, VCAM-1, and E-selectin. DHMEQ also inhibited TNF-α-induced mononuclear cell-HUVEC adhesion. The effect of DHMEQ was more prominent when the cells were under flow. Thus, DHMEQ acted on vascular endothelial cells to inhibit mononuclear cell adhesion in vitro and ameliorated the atherosclerosis model in vivo.

The amniotic membranes are directly in contact with the amniotic fluid and restrict fluid flux during pregnancy. These membranes usually rupture in the process of labor. Apoptosis of amniotic epithelial cells may cause disruption of tight junctions, resulting in rupture of the membranes. Kobayashi, Yasui, et al. studied the molecular mechanism underlying apoptosis of the amniotic epithelium of the mouse using DHMEQ [75]. The number of activated macrophages expressing high levels of iNOS and TNF-α increased in the amniotic fluid towards later stages. TNF receptor type 1 (TNFR1) also became detectable. After that, apoptosis of amniotic epithelial cells began, suggesting that TNF-α/TNFR1 signaling may initiate apoptosis. Both TNF-α/TNFR1 antagonist and DHMEQ inhibited TNF-α production in macrophages and amniotic apoptosis in vivo. These results indicate that amniotic apoptosis is induced by the NF-κB-dependent TNF-α production in macrophages and activation of TNFR1 signaling in the amniotic epithelial cells. The levels of apoptotic cells also increase in the case of preterm rupture of the fetal membrane. Therefore, DHMEQ may be useful to prevent baby loss caused by the preterm rupture of the amniotic membrane.

Thus, IP administration of DHMEQ ameliorated sepsis, asthma, atherosclerosis, and amniotic membrane preterm rupture models. It may be useful for these diseases and conditions in the future.

The anti-inflammatory activities of DHMEQ in vivo are summarized in Table 1. It is interesting that NF-κB activity is involved in the mechanism of so many disease progressions.

## 5. Possible Mechanism of Action for Intraperitoneal DHMEQ Therapy

DHMEQ effectively enters the cells to inhibit NF-κB when added to the cultured cells [19]. In the case of topical application to the skin or subcutaneous injection near the inflammatory site, DHMEQ is considered to reach the inflammatory sites and act directly there, as shown in Figure 5.

**Figure 6:**
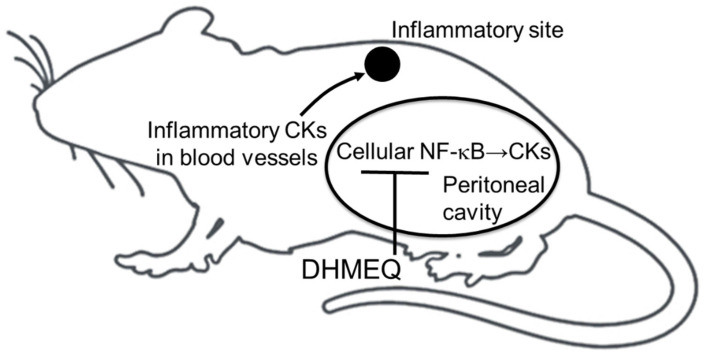
Possible mechanism for anti-inflammatory activity of DHMEQ by IP administration. DHMEQ acts only in the peritoneal cavity. CK, cytokines.

However, when DHMEQ is administered into the peritoneal cavity, it appears difficult for the drug to reach the inflammatory sites. When injected intravenously or intraperitoneally, the blood concentration of DHMEQ did not increase [76]. It is because DHMEQ is easily inactivated in the blood. DHMEQ is quickly absorbed and inactivated by the blood cells. Instead, the IP concentration of DHMEQ is kept for at least 60 min over the effective concentrations. Therefore, it is likely that DHMEQ only acts in the peritoneal cavity after the injection to inhibit the inflammatory cell activity there. However, the blood concentration of inflammatory cytokines can be reduced by IP administration [77]. A possible mechanism of action for the anti-inflammatory activity by IP administration of DHMEQ is illustrated in Figure 6. DHMEQ should inhibit inflammatory cytokine production in the peritoneal cavity, which reduces blood concentration of inflammatory cytokines. Lower cytokine concentration is likely to inhibit peripheral inflammation. The peritoneal cavity appears to be a special place for macrophages. Vernon–Roberts studied the traffic of macrophages in the body using radioactively labeled macrophages in mice. The peritoneal cavity was found to be an especially important space for macrophages. Macrophages usually reside in the peritoneal cavity, but in case of inflammation, they leave there and move to areas of inflammation [78]. This finding can explain why the blood concentration of inflammatory cytokines decreases by the inactivation of peritoneal macrophages.

For the clinical application of IP therapy, DHMEQ should inhibit NF-κB and cytokine release in human peritoneal cells. Soninska, Breborowicz, et al. reported that DHMEQ suppressed inflammatory reactions and collagen production in primary cultured human peritoneal cells [79].

The anti-inflammatory activity of DHMEQ is thus based on the unique mechanism in the case of IP administration. DHMEQ neither enters the blood circulation nor reaches the inflammatory site. This mechanism can explain why DHMEQ shows no side effects in all the animal experiments thus far. The anticancer activity of DHMEQ is dependent on the same mechanism [80].

## 6. Discussion and Future Perspective

Highly purified DHMEQ can be synthesized from inexpensive compounds in five steps, which would be advantageous to its development. The mechanism of inhibition is unique since DHMEQ binds to specific cysteine residues of NF-κB molecules. Since DHMEQ covalently binds to NF-κB components, the inhibition is irreversible. Irreversible inhibition would result in effective inhibition in vivo, although DHMEQ is short-lived in the body.

DHMEQ has been widely used to suppress various inflammatory disease models. Topical application inhibited various atopic dermatitis and rheumatoid arthritis models. IP administration inhibited various inflammatory disease models and graft rejections. These results suggest that NF-κB is involved in the etiology of many diseases.

NF-κB is an attractive target for anti-inflammatory and anticancer drugs. However, no NF-κB inhibitor has been developed for clinical use so far. This is because it is difficult to avoid toxicity. Stable NF-κB inhibitors should inhibit the blood cell differentiation and lower the stability of tissues. On the other hand, there have been no toxic observations thus far in animal experiments with DHMEQ. This is because of the instability of DHMEQ in the body. It is easily degraded in the presence of blood cells. Instability in the blood is certainly disadvantageous for its development into drugs since intravenous administration is not applicable. At present, DHMEQ ointment is being developed in the industry. In this case, DHMEQ irreversibly inhibits NF-κB of inflammatory cells in the skin, and after that, the chemical is degraded.

Until the discovery of antibiotics, immunity was always advantageous to avoid disease in humans. However, at present, excess immunity often causes inflammatory diseases, including autoimmune and chronic inflammatory diseases. IP administration of DHMEQ is essentially an immunosuppressive therapy since it was shown to act like tacrolimus in organ transplantation experiments. IP administration of DHMEQ showed anti-inflammatory activities without toxicity in many animal experiments. Although IP injection is not popular for clinical use, IP administration of DHMEQ may become a useful therapy for a wide range of inflammatory diseases in the future.

In the future, DHMEQ will still be useful to study the mechanism of diseases and physiological phenomena. DHMEQ can inhibit both canonical and non-canonical NF-κBs. When DHMEQ is added to the cell or tissue culture experiments, it inactivates 4 NF-κB components, p65, p50, RelB, and cRel. It would be difficult to inactivate these proteins together by si-RNA knockdown or CRISPR-Cas9 knockout techniques. Therefore, DHMEQ is recommended for use in cell or tissue culture experiments for mechanistic study. Highly purified DHMEQ is available in the corresponding author’s lab.

## Figures and Tables

**Table 1:** Anti-inflammatory activity on in vivo disease models.

Disease or Treatment	Key Findings	Route	References
Atopic dermatitis	Amelioration of inflammatory score	Ointment	[29,30,31,32]
Rheumatoid arthritis	Amelioration of arthritic score	IP	[8]
Rheumatoid arthritis	Inhibition of paw swelling and bone destruction	SC	[38,39,41]
Anti-phospholipid disease	Inhibition of thrombus formation	IP	[45]
Uveitis	Reduction of infiltrating cells	IP	[46,47]
Retinal inflammation	Reduction of infiltrating cells and preservation of rhodopsin	IP	[49,50,51,52]
Corneal inflammation	Inhibition of angiogenesis	IP	[54]
Inflammatory bowel diseases	Amelioration of inflammatory score	IP	[55,56,57,58,59]
Intestinal ischemia/reperfusion	Amelioration of mucosal damage	IP	[60]
Kidney inflammation	Preservation of creatinine clearance and reduction of fibrosis	IP	[61,62,63,64]
Heart transplantation	Prolongation of graft survival	IP	[65,66]
Islet transplantation	Prolongation of graft survival	IP	[67,68,69]
Bone marrow transplantation	Amelioration of GVHD	IP	[70]
Sepsis	Prolongation of survival	IP	[71]
Asthma	Amelioration of airway remodeling	IP	[72]
Atherosclerosis		IP	[73]
Premature birth		IP	[75]

## Data Availability

No new data was created or analyzed in this study. Data sharing is not applicable to this article.
